# Supervised and home-based physical exercise in patients newly diagnosed with multiple myeloma—a randomized controlled feasibility study

**DOI:** 10.1186/s40814-019-0518-2

**Published:** 2019-11-12

**Authors:** Rikke Faebo Larsen, Mary Jarden, Lisbeth Rosenbek Minet, Ulf Christian Frølund, Niels Abildgaard

**Affiliations:** 1 Department of Physiotherapy and Occupational Therapy, Zealand University Hospital, Sygehusvej 10, 4000 Roskilde, Denmark; 2 Department of Clinical Research, University of Southern Denmark, Campusvej 55, 5230 Odense M, Denmark; 3 OPEN, Odense Patient data Explorative Network, Odense University Hospital, J.B. Winsløwvej 9A, 5000 Odense, Denmark; 4 Department of Hematology, Copenhagen University Hospital, Rigshospitalet, Blegdamsvej 9, 2100 Copenhagen, Denmark; 5 Department of Public Health, Faculty of Health and Medical Sciences, University of Copenhagen, Blegdamsvej 3B, 2200 Copenhagen, Denmark; 6 Department of Rehabilitation, Odense University Hospital, J.B. Winsløws Vej 4, 5000 Odense C, Denmark; 7 Center for Applied Research, UCL University College, Niels Bohrs Allé 1, 5230 Odense M, Denmark; 8 Department of Hematology, Zealand University Hospital, Sygehusvej 10, 4000 Roskilde, Denmark; 9 Department of Hematology, Odense University Hospital, Kløvervænget 10, 5000 Odense, Denmark; 10 The Academy of Geriatric Cancer Research (AgeCare), Odense University Hospital, J.B. Winsløws Vej 4, 5000 Odense, Denmark

**Keywords:** Physical exercise, Multiple myeloma, Bone disease, Feasibility, Safety

## Abstract

**Background:**

The study evaluated the feasibility and safety of the exercise intervention and physical test procedures of our ongoing randomized controlled trial, examining the effect of physical exercise in newly diagnosed patients with multiple myeloma.

**Methods:**

Patients are randomized 1:1 to a control group (usual care) or an intervention group (usual care and exercise) by block randomization with stratification of planned treatment, WHO performance status, and study site. The exercise intervention consists of eight supervised exercise sessions combined with home-based exercise over a 10-week period. Bone disease is systematically evaluated to determine limitations regarding physical testing and/or exercise. Feasibility outcome measures were study eligibility, acceptance, and attrition, and furthermore attendance, adherence, tolerability, and safety to the exercise intervention. Additionally, test completion, pain, and adverse events during the physical test procedures were evaluated. Outcome assessors were blinded to allocation.

**Results:**

Of 49 patients screened, 30 were included. The median age was 69 years, range 38–90, 77% were males, and 67% had bone disease. Study eligibility was 82%, acceptance 75%, and attrition 20%. Attendance at supervised exercise sessions was 92%, and adherence to supervised exercise sessions and home-based exercise sessions was 99% and 89%, respectively. No serious adverse events attributed to exercise or physical tests were reported. All patients completed the physical tests, except for two patients, where physical test procedures were modified due to bone disease.

**Discussion:**

The exercise intervention and physical test procedures were feasible and safe in patients with multiple myeloma, even in older patients with multiple myeloma and in patients with myeloma bone disease.

**Trial registration:**

ClinicalTrials.gov. ID NCT02439112. Registered on May 7, 2015.

## Background

Physical exercise in patients with hematological cancer has been shown to be feasible and safe and yielding benefits for aerobic capacity, muscle strength, quality of life (QoL), psychosocial wellbeing, treatment-related symptoms, fatigue, and body composition, before, during, and after stem cell transplantation [1–4]. However, exercise research in hematological malignancy is rather sparse [5, 6], having been carried out in specific hematological diagnoses such as acute leukemia [1]. Few exercise studies have been conducted in patients with multiple myeloma (MM), recently reviewed by Gan et al. [7].

MM is a plasma cell cancer in the bone marrow that primarily affects older adults. The incidence and prevalence have increased as the aging population continues to grow, and survival has improved due to advancements in medical treatments [8–11]. In Europe, the incidence of MM is 5.72 per 100,000, and the median age at diagnosis is 68 years [9]. At the time of diagnosis, most patients have a symptomatic disease that requires treatment.

Younger, fit patients (< 65–70 years) are treated with bortezomib-based induction treatment followed by high-dose chemotherapy with stem cell support (HDT-SCT) [12]. Older patients or patients with comorbid conditions receive less intensive, yet still effective treatments that include the proteasome inhibitor bortezomib and/or the immunomodulatory agent lenalidomide [13–15]. Bone disease with osteopenia, pathological fractures, and typically “punched out” lytic lesions are hallmarks of the disease and are present in approximately 80% of the patients at the time of diagnosis and even more during the course of the disease [16]. The bone disease is caused by myeloma-induced increased bone degradation by osteoclasts and inhibited the formation of new bone matrix by osteoblasts [17]. Painful bone lesions may be treated with radiation therapy, and all patients receive intravenous bisphosphonates to reduce the risk of progressive bone disease, pain, and fractures [18, 19]. Anemia is present in 70–80% of the patients [16, 20]. Patients with MM experience more symptoms and more severe symptoms than patients with other hematological diseases, negatively affecting QoL [21]. Due to the frequent and potentially serious bone involvement, and because MM is a cancer in the older population, the potential role of exercise needs to be investigated separately in patients with MM.

Three randomized controlled trials [22–24] and one single-arm pilot study [25] investigating the effect of exercise in patients with MM have been conducted and summarized in the review by Gan et al. [7]. The exercise interventions comprised stretching, aerobic exercise, and strength resistance exercises [22–25], lasted between 18 and 26 weeks, and started either approximately 10 weeks after the start of induction [22–24] or after HDT-SCT [25]. The studies found exercise to be feasible and safe, whereas efficacy data showed mixed results. However, studies that intervene at the time of diagnosis and start of active anti-myeloma therapy are lacking, as are studies that include older patients who comprise the majority of patients newly diagnosed with MM. Thus, the effectiveness of participation in exercise programs remains unclear for patients with MM.

Gan et al.’s exercise recommendations for patients with MM suggest that exercise should be individually adjusted, taking the severity of the disease and the aggressiveness of the treatment into consideration to prevent or minimize physical deterioration [7].

In 2015, we initiated a randomized controlled trial (RCT) to investigate the efficacy of early initiated, individualized physical exercise intervention, combining supervised exercise sessions and home-based exercise sessions and physical activity in patients newly diagnosed with MM. The RCT is still ongoing. The aim of the current study is to evaluate the feasibility and safety of the exercise intervention and physical test procedures. The feasibility of participation is evaluated by eligibility, acceptance, and attrition to the study. Feasibility and safety of the exercise intervention are evaluated by attendance, adherence, tolerability, attrition, and adverse events (AEs). Feasibility and safety of the test procedure were evaluated by completion, registration of pain, and AEs. We have used the CONSORT 2010 statement: extension to randomized pilot and feasibility trials [26].

## Methods

### Study design, patient recruitment, and procedures

The RCT is a two-center study, with blinded outcome assessors, carried out at the Departments of Hematology at Zealand University Hospital, Roskilde, and Odense University Hospital in Denmark. A total of 102 patients will be included for efficacy evaluation in the RCT. The primary objective of the RCT is muscle strength of the knee extensor muscles measured by dynamometer [27], and secondary objectives are physical measures (30 s Sit-to-Stand Test, grip strength, Six-Minute-Walk Test), level of physical activity (by accelerometers), QoL (EORTC-QOLQ-C30 and EORTC-QLQ-MY20), pain (Brief Pain Inventory, short version), and bone disease (DEXA-scans and markers of bone metabolism markers). Outcomes are assessed after 11 weeks, 6 months, and 12 months.

Patients are consecutively screened for eligibility at the time of diagnosis by the hematologists at each site, based on inclusion and exclusion criteria. Patients > 18 years newly diagnosed with MM planned for HDT-SCT or less intensive treatment regimens are eligible. The patient must speak and understand Danish. Exclusion criteria are spinal cord compression, unstable vertebral fracture (SINS score > 12) [28], untreated cardiac failure or untreated cardiac arrhythmia, severe chronic cardiac failure (NYHA 3–4), other severe comorbidities that would not permit physical exercise, and psychological or psychiatric disorders. Informed consent is obtained from all individual participants included in the study.

The hematologist performs a systematic assessment of the impact of bone disease to determine restrictions regarding physical tests or exercise. This assessment is based on radiographs or computed tomography of the skeleton, and captured site, size of osteolytic lesions, and if applicable, time since fracture, moreover the degree of pain. Bone destructions are assessed using the principles of Mirels´ scoring system [29]. Restrictions of not performing the static knee extensor strength and 30 s Sit-to-Stand Test is given if a fracture is detected in the femoral bone, if the osteolysis has a size of over two-third/involving compacta, or if the size is between one third to two third accompanied by any kind of pain, or finally, if there is femoral bone destruction with moderate or functional pain. Restriction to test of knee extensor strength is only for the affected side. The same assessment is applied for exercise restrictions, and the humeral bones are assessed in the same way. Furthermore, the pelvis, costae, thoracic, and lumbar spine are assessed. Pelvis restriction is given if there is fracture or osteolysis (> 2 cm of the acetabulum or two third of rami). New fractures (less than 6 weeks) of the costae or vertebral bodies will result in restrictions, or a former fracture accompanied by any kind of pain will also lead to restriction. Exercise restrictions followed the resistance and flexibility principles by Galvão et al. [30], which generally means that patients do not use weights in the strengthening exercises for the involved site and movements are restricted at the involved site, e.g., rotation of the spine.

Patients are tested at baseline within 1 week after the start of active anti-myeloma treatment. Assessment is conducted by physiotherapists, who have received a structured introduction to the test procedure. Hereafter, patients are randomized 1:1 to an intervention group (IG) or control group (CG). Block randomization and stratification according to treatment (planned HDT-SCT versus (vs.) non-intensive treatment), WHO performance status (PS 0–1 vs. PS ≥ 2) [31], and study site are performed. The randomization procedure follows a random allocation list, which is made prior to study commencement. The randomization is conducted by a project nurse who is not part of the study group, and the randomization list is only available to the project nurse, and thus, outcome assessors are blinded to allocation.

This feasibility study evaluated the first 30 included patients in the period from June 22, 2015, to June 30, 2016. This is considered as an adequate sample size because of the nature and aim of this feasibility study [32].

### Control group

**Fig. 1:**
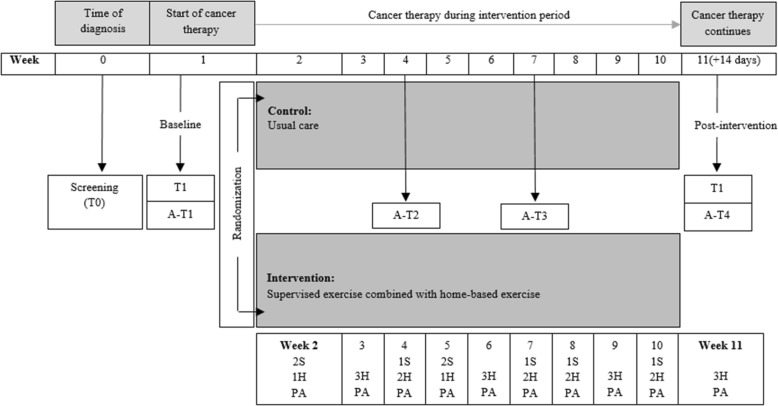
Overview of the randomized controlled feasibility study including intervention and physical measurements. T0; Time 0 corresponding to time of screening. T1; Time 1 corresponding to physical tests at baseline test. T2: Time 2 corresponding to physical tests post-intervention. A-T1; Activity-Time 1 corresponding to accelerometer measures at baseline. A-T2; Activity-Time 2 corresponding to accelerometer measures at week 4. A-T3; Activity-Time 3 corresponding to accelerometer measures at week 7. A-T4; Activity-Time 4 corresponding to accelerometer measures post-intervention. 1S and 2S; Supervised exercise session one or two times weekly, respectively. H1, H2, and H3; Home-based exercise session one, two, or three times weekly, respectively. PA; Physical activity taking place the remaining 4 days, where exercise session is not conducted

The CG receives usual care, which consists of written information on the importance of being physically active, suggestions on how to remain physically active, and ergonomic guidance on how to lift and perform transfers properly from a lying to sitting position. Written information is given to the patient, by a study physiotherapist or a nurse, during the second week after the start of treatment. Usual care could (if needed) also include a physician-ordered rehabilitation plan, prescribing exercise for the patient in the municipality, see Fig. 1.

### Intervention group

In addition to usual care, the patient is instructed to do the exercise program three times/week and to be independently physically active for 30 min per day, the other 4 days of the week. The exercise intervention is designed to meet the Danish recommendations for persons > 65 years and for patients with cancer including being physically active 30 min a day for at least 10 continuous minutes at moderate intensity [33, 34]. Further, at least two times a week, the activity must be of high intensity and include strengthening exercises and stretching [33, 34]. The Danish recommendations are in accordance with international guidelines [35–37].

The patient receives careful instruction regarding the exercise intervention and a booklet with a description of the exercises. Instructions are carried out by a study physiotherapist who received careful and structured introduction to the exercise intervention. The exercise program is conducted three times weekly, and it fluctuates between being conducted under supervision or unsupervised at home. Furthermore, the patient is expected to be physically active, the remaining 4 days, see Table 1. In total, there are eight supervised exercise sessions during the 10-week intervention period, starting 1 week after diagnosis, see Fig. 1. The interval between the supervised exercise sessions varies, because the sessions are planned according to the patients’ treatment plan to minimize the number of visits to the hospital. The patient receives an exercise diary to document adherence to the intervention, and the study physiotherapist uses the diary as a pedagogical and motivational planning tool. Each supervised exercise session lasts for 1 h + 15 min and consists of warm-up, aerobic exercise, strengthening exercises, and static stretching exercises, see Table 1.

**Table 1:** Exercise intervention; mode, intensity, duration, and progression

Mode	Intensity	Duration per session	Progression
Exercise program 3 times/week			
Warm up	10–11 RPE	5 min	
Aerobic exercise	12–13 RPE	20 min	↑ intensity to 14–16 RPE
Strengthening exercise	3 sets of 12–15 reps	30–45 min	↑ weight to 3 sets of 10–12 reps
Five exercises for the lower extremities^a^			
Three exercises for the upper extremities^b^			
One exercise for truncus^c^			
Stretching	30 s static	5 min	–
Three muscle groups of the lower extremities^d^			
Physical activity 4 times/week preference of the patient	12–13 RPE	30 min at least for 10 continuous min	14–16 RPE (is a possibility, but not a standard)

Aerobic exercise: If not possible to do aerobic exercise for 20 min on the stationary bike during the supervised session, the progression is an increase in total time (up to 20 min)

*RPE* rate of perceived exertion, *Reps* repetitions

^a^Knee extension in sitting position, knee flexion in standing position, hip extension in a prone position, toe raising in standing position, and knee bent or raise from the chair

^b^Arm lift in frontal plane or circulation of shoulders in standing position, elbow extension in a supine position, and elbow flexion in standing or sitting position

^c^Static in supine with knees bent or supine position with knee bent and lift of foot with press from the opposite hand

^d^Femoral muscles (standing position), hamstring muscles (standing or sitting position), and calf muscles (standing in front of the wall)

### Outcome measures

Data were collected at four time points; T0: time of diagnosis (screening for eligibility), T1: baseline (pre-intervention), Ti: during intervention (weeks 1–10) and T2: post-intervention (weeks 11–13), see Fig. 1.

Outcomes measures were as follows:

1. Feasibility of participation at T0: eligibility, acceptance, and attrition rates were registered as well as reasons for non-eligibility and decline.
2. Demographic and medical data at T1: age, gender, PS, plan of treatment, and bone disease.
3. Feasibility and safety of the intervention at Ti: attendance, adherence, tolerability, attrition, and AEs. The reason for and the number of time of dropouts were registered. Attendance, adherence, tolerability, and safety of the supervised exercise sessions were obtained by intervention logs and documented by the study physiotherapist. Adherence to home-based exercise sessions was documented in an exercise diary. Safety, i.e., AEs, during and between supervised exercise sessions were recorded by observation (during sessions) and questioning patients at each of the supervised sessions. Further, patients documented AEs in their exercise diary.
4. Feasibility and safety of physical tests at T1 and T2 and of accelerometer measurements at A-T1, A-T2, A-T3, and A-T4. The strength of lower extremities was measured by two tests; Static knee extension strength test by dynamometer [23, 38, 39] and 30 s Sit-to-Stand-Test [39, 40]. Upper body strength was measured by grip strength, using a hand-held dynamometer [27, 39]. Submaximal aerobic capacity was measured by Six-Minute-Walk Test [23, 41, 42]. Feasibility was measured by completion rates and safety by recording of pain, if any. Other AEs were recorded by the study physiotherapist.

### Statistical analysis

Descriptive statistics were conducted using simple report data from the project database in REDCap provided by Open Patient data Explorative Network (OPEN), Odense University Hospital, Odense, Denmark. The analysis was based on intention to treat. Rates of eligibility, acceptance, attrition, attendance, and adherence are presented in numbers and percentages, as well as completion rates of physical tests. Furthermore, the number of patients perceiving pain or AEs was recorded. Medical and demographic data were collected and presented for all included patients and for each group separately (IG and CG).

## Results

### Demographics and medical characteristics

The baseline characteristics of the participants are summarized in Table 2. The median age was 69 years (range 38–90), 46% of the patients were above 70 years, and 75% were men. Sixty-seven percent had bone disease, and half of them were assessed to have restrictions for tests or exercise. The two groups (IG vs. CG) were comparable in age, gender, PS, and planned treatment. Bone disease in the intervention group was higher than in the control group, but not in whether the bone disease led to any restrictions regarding tests or exercise.

**Table 2:** Patient characteristics

Patient characteristics	Total *N* = 30	IG *n* = 17	CG *n* = 13
Age (years)			
Mean (SD)	68 (12.2)	69 (9.7)	67 (15.3)
Median (range)	69 (38–90)	68 (48–82)	70 (38–90)
Age groups, years (*n* (%))			
≤ 49	3 (10)	1 (6)	2 (15)
50–59	4 (13)	2 (12)	2 (15)
60–69	9 (30)	7 (41)	2 (15)
70–79	10 (33)	5 (29)	5 (38)
80–89	3 (10)	2 (12)	1 (8)
≥ 90	1 (3)	0 (0)	1 (8)
Gender (*n* (%))			
Male	23 (77)	14 (82)	9 (69)
Female	7 (23)	3 (18)	4 (31)
WHO performance status (*n* (%))			
0–1	25 (83)	13 (77)	12 (93)
≥ 2	5 (17)	4 (24)	1 (8)
Planned treatment (*n* (%))			
HDT-SCT^a^	18 (60)	10 (59)	8 (62)
Not HDT-SCT	12 (40)	7 (41)	5 (38)
Bone disease, in general (*n* (%))			
No	10 (33)	3 (18)	7 (54)
Yes	20 (67)	14 (82)	6 (46)
Bone disease with restriction for tests or exercise, *n* = 20 (*n* (%))			
No	10 (50)	9 (64)	1 (17)
Yes	10 (50)	5 (36)	5 (83)

^a^*HDT-SCT* high-dose therapy with stem cell transplantation

### Feasibility and safety

#### Eligibility, acceptance, and attrition

**Fig. 2:**
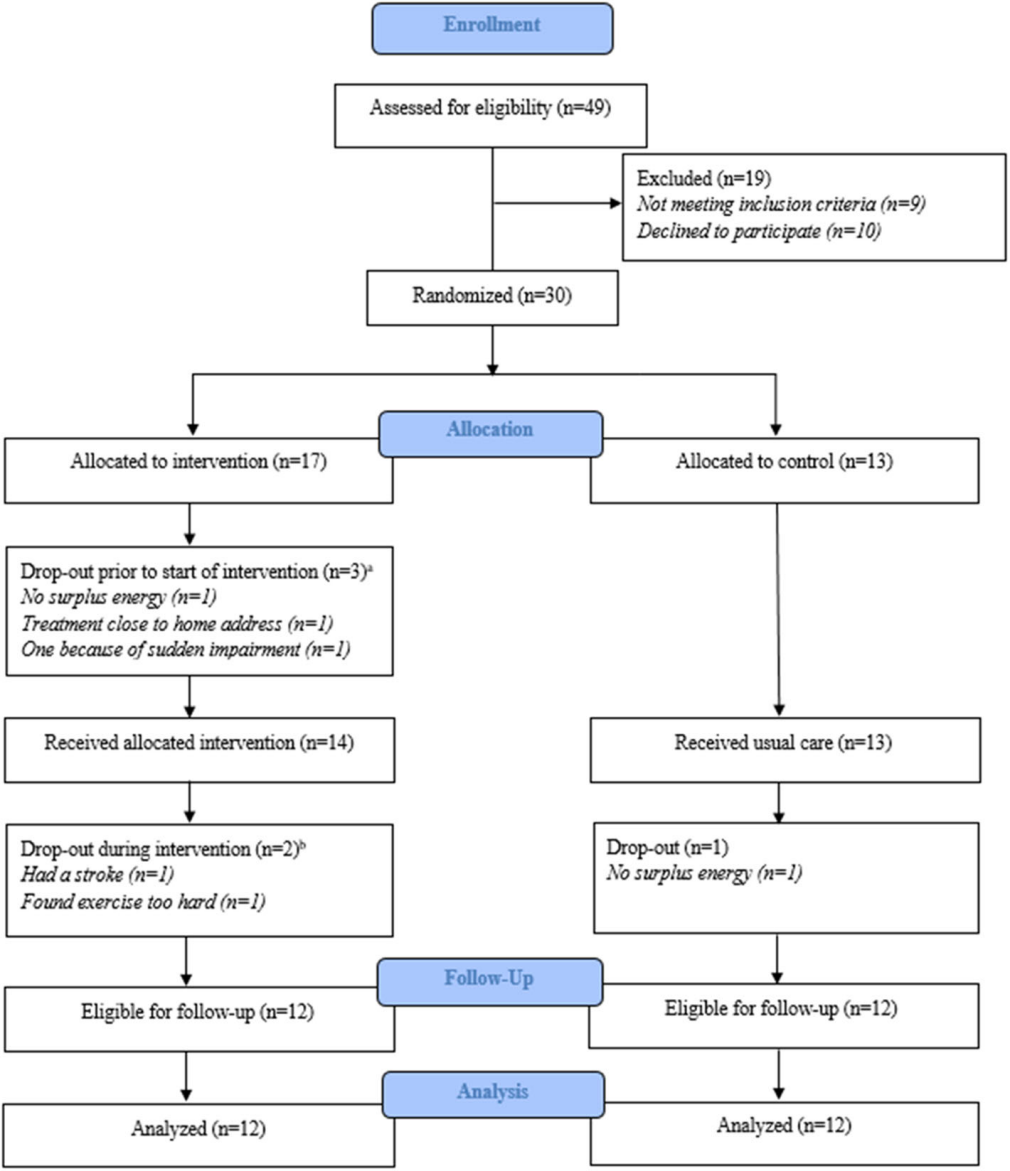
Flowchart based on the CONSORT 2010 flow diagram [26]. ^a^One patient was tested at baseline (T1). Two patients dropped out before performing the baseline test, which is considered as a randomization failure. ^b^One patient dropped out before session 4 and one before session 8

Of 49 patients screened at T0, 40 met the inclusion criteria (82% eligibility). Reasons for non-eligibility were comorbidity (*n* = 3), spinal cord compression (*n* = 2), bilateral involvement of the femoral bone (*n* = 3), and immobility because of pain (*n* = 1). Of the 40 eligible patients, 30 accepted participation (75% acceptance rate) and ten patients declined (25%). Reasons for the decline were lack of energy (*n* = 4), not interested in exercise (*n* = 2), and unknown (*n* = 4). Of the 30 patients included, six participants dropped out after inclusion (20% attrition); from IG, five out of 17 participants (29%) and from CG, one out of 13 participants (7%). From IG, two dropped out prior to baseline test (T1) (lack of energy (*n* = 1), sudden impairment (*n* = 1)), and furthermore, there was a randomization failure in these two cases, since they were randomized before T1. One dropped out prior to the start of exercise intervention (the patient had the possibility of receiving anti-myeloma treatment closer to home). Two dropped out during the intervention period (due to stroke (*n* = 1) and due to experiencing exercise as being too strenuous (*n* = 1)). Dropouts took place before the fourth and the eighth sessions, respectively. From CG, one participant dropped out because of a lack of energy to participate in the study, see Fig. 2.

#### Attendance at supervised exercise sessions

In total, 12 participants out of 14 participants (86%) who started intervention completed the full intervention, and 11 out of 12 participants (92%) attended all supervised sessions. The one participant who did not attend all sessions participated in seven out of eight sessions, and the one session was canceled by the participant for private reasons, see Table 3.

**Table 3:** Adherence to the intervention and the individual components of the intervention, and adverse events

	IG *n* = 12*	Comments
Adherence to supervised exercise session		
Patients who completed (*n* (%))	11 (92%)	One participant canceled one session because of the condition.
Sessions completed (*n* (%))^a^	95 (99%)	
Adjustments of the exercise program		
Progression of exercise program (*n* (%))	4 (33%)	
Regression of exercise program (*n* (%))	1 (8%)	
No progression or regression (*n* (%))	0 (0%)	
Both progression and regression (*n* (%))	7 (58%)	
Adherence to home-based exercise sessions (*n* (%))^b^	203 (89%)	
Adherence to physical activity^c^	405 (94%)	
Diary registration (*n* (%))		
All weeks	10 (83%)	
Some weeks	2 (17%)	
No weeks	0 (−)	
Adverse events (*n*)	2	Dizziness (*n* = 1), symptoms of pain (*n* = 1). All non-serious adverse events.
Consequences of the adverse events		
None	0	
Discontinuation of the supervised exercise session (*n*)	2	

*Data is based on participants who completed the intervention for the whole intervention period (*n* = 12)

^a^Out of 96 possible sessions (eight sessions for each participant)

^b^Out of 228 recommended sessions based on a period of 9 weeks

^c^Out of 432 recommended sessions based on a period of 9 weeks

#### Adherence, tolerability, and safety

The adherence rate of the supervised exercise sessions was 99%. Two patients discontinued one supervised session each, due to non-serious AEs; symptoms of pain (*n* = 1) and dizziness (*n* = 1), see Table 3. None of the AEs were found to be related to testing or exercise. Importantly, no patients experienced pathological fractures during testing or exercise.

Adherence to home-based exercise sessions was 89%, and 94% out of the recommended number of days with physical activity were completed. Eighty-three percent of the participants had complete diary registration.

All physical tests were tolerated and safe. All participants, except one, were able to complete the knee extensor strength test (primary outcome), at least in one leg. We lack information about the reason for the missed knee extensor strength test in the one participant.

At T1 and T3, 82% and 88%, respectively, completed the knee extensor strength test in both legs. Test completion of the secondary outcomes was 100%, except for two participants, who did not complete the 30 s SST, see Table 4. The completeness of data from accelerometers was 92–96%. We had apparatus failure (*n* = 2) at A-T4, and in one case at A-T3, we were not able to detect the reason for incomplete data. Missing data at A-T1 were unknown, and at A-T2, the participant did not wear the accelerometer. There were no AEs, e.g., skin irritation.

**Table 4:** Patients who performed the physical tests and worn accelerometers at the investigated times

Physical tests	T1^*^ n = 28	T2^#^ *n* = 24	A-T1^*^ n = 28	A-T2 *n* = 24	A-T3 *n* = 24	A-T4^#^ *n* = 24
Knee extensor strength test (*n* (%))						
Both legs tested	23 (82)	21 (88)				
Only one leg tested because of bone restriction	2 (7)	2 (8)				
Only one leg tested because of patient inability	0 (−)	1 (4)				
Only one leg tested without explanation	2 (7)	0 (−)				
Not done	1 (4)	0				
Pain during test^a^	4 (14)	5 (21)				
Adverse events	0	0				
Grip strength test (*n* (%))						
Patients who performed the test	28 (100)	24 (100)				
Not done	0 (−)	0 (−)				
Pain during test^b^	5 (18)	3 (13)				
Adverse events	0	0				
30 s Sit-to-Stand Test (*n* (%))						
Patients who performed the test	26 (93)	24 (100)				
Not done	2 (7)	0 (−)				
Pain during test^c^	5 (19)	1 (4)				
Adverse events	0	0				
Six-Minute-Walk Test (*n* (%))						
Patients who performed the test	28 (100)	24 (100)				
Not done	0 (−)	0 (−)				
Pain during test^d^	8 (29)	5 (21)				
Adverse events	0	0				
Accelerometers (*n* (%))						
Worn, complete data			27 (96)	23 (96)	23	22
Worn, incomplete data			0 (−)	0 (−)	(96)	(92)
Not worn/missing			1 (4)	1 (4)	1 (4)	2 (8)
Adverse events			0 (−)	0 (−)	0 (−)	0 (−)

^a^Pain during test of knee extensor strength test

At T1; related to equipment (*n* = 2), knee pain (*n* = 1), undescribed (*n* = 1) S

At T3; related to equipment (*n* = 2), back pain (*n* = 1), minor leg pain (*n* = 1), missing (*n* = 1)

^b^Pain during test of grip strength

At T1; sternum (*n* = 1), clavicular (*n* = 2), breast muscle (*n* = 1), sternum and costae (*n* = 1)

At T3; fingers (*n* = 1), costae (*n* = 1), known pain (*n* = 1)

^c^Pain during 30 s Sit-to-Stand Test

At T1; knee pain (*n* = 1), back pain (*n* = 2), scapula and sternum (*n* = 1), thorax (*n* = 1)

At T3; back pain (*n* = 1)

^d^Pain during Six-Minute-Walk Test

At T1; thorax (*n* = 1), sternum (*n* = 1), scapula and sternum and right hip (*n* = 1), thorax and dyspnea (*n* = 1), toe (*n* = 1), missing (*n* = 2)

At T3; hip muscle pain (*n* = 1), reaction from the thigh (*n* = 1), back pain (*n* = 1), Achilles tendon (*n* = 1), lower extremity (*n* = 1)

^*^T1 and A-T1 correspond to the same time point (baseline)

^#^T2 and A-T4 correspond to the same time point (post-intervention)

## Discussion

This study examined the feasibility and safety of an early initiated, individualized physical exercise intervention, combining supervised exercise sessions and home-based exercise sessions in combination with physical activity in patients newly diagnosed with MM. Our main finding was that the exercise intervention and physical test procedures were feasible and safe.

We succeeded to include a broad group of patients, including older patients planned for less intensive treatment than HDT-SCT. In only one former study in patients with MM in stable phase, and either off treatment or on maintenance therapy [25], patients were included regardless of whether they had undergone a HDT-SCT or other chemotherapeutic treatments. However, only 8% had not undergone HDT-SCT, compared to 40% in our study. The median age was 61 years, range 46–74 years, compared to 69 years, range 38–90 years in our study. The median age of 69 years indicates that concerning age our cohort is representative for the general MM population.

The eligibility and acceptance rates in our study are in accordance with results from other studies [25, 43], even though our study started recruitment at an earlier stage and with the inclusion of older patients. This indicates that participants found exercise relevant at the time of diagnosis, as well as during the recovery phase (6–14 weeks after first-line HDT-SCT) [43] and in the stable plateau phase [25].

Forty-nine patients were screened for participation in the study during the first year. This was fewer than expected according to the Danish MM Registry, which about 75 patients with newly diagnosed MM should have been diagnosed at the two departments within 1 year [44]. Thus, approximately one third of the newly diagnosed patients with MM were not assessed for eligibility. There are several possible explanations for this; some of the most likely are disease presentation with severe complications (including severe infections), need of hemo-dialysis, and severe immobilization due to bone pain. Another reason is that some hematologists simply forgot to screen and offer participation to some patients. Probably, the included patients are skewed according to the severity of disease and have fewer complications at diagnosis than the general MM population. Twenty-five percent of the eligible patients declined to participate. The time of diagnosis is a sensitive time for the patient with a large information burden, and some patients are anxious and have difficulty coping with their situation. We included fewer female patients than expected, which is not a finding supported by the literature [45].

The attrition rate in IG (29%) is within, but in the high end of, the range that has been observed in other studies (4%–29%) [22, 23, 25, 43]. The attrition rate in the CG (7%) is lower than in other studies, where attrition rates ranged from 15 to 30% [22, 23, 43]. The four-times higher attrition rate in IG than in CG can partly be explained by randomization failure. By following a stricter randomization procedure, we expect more equal attrition rates in the larger RCT, although the intervention itself might play a role. Thus, exercise intervention at the time of diagnosis is feasible for most, but not all patients with MM. However, it is noteworthy that the attendance and the adherence were relatively high. In total, 24 out of 30 included patients (80%) were available for analysis, which is informative for us regarding dropouts in the RCT.

The studies with the lowest overall attrition (regardless of group assignment) [25, 43] took place either in the recovery phase or stable plateau phase. Nevertheless, attrition during active anti-myeloma treatment can be expected to be higher. The overall attrition in our study (20%) is within the range of other studies conducted during treatment (11–42%) [22–24, 43].

Our attendance rate to supervised exercise sessions was higher than the rates in other studies with supervised sessions [25, 43]*.* Our more favorable attendance rate may be because we strive to plan the sessions on the same days as the medical visits at the hospital, contrary to, e.g., exercise in a physiotherapy practice [43].

Adherence to supervised exercise sessions was 99%. Discontinuation was a minor issue, and no serious AEs related to physical exercise or testing were registered. Adherence to home-based exercise sessions was 89%, which is in accordance with the adherence of 86% in another study with a mixed intervention (supervised and home-based) [25].

Importantly, we observed no pathological fractures, even though we intervene at a very early stage. The same safety findings were reported in other studies of exercise in patients with MM [23–25].

The completion rates of the physical tests were high, not least of the primary outcome (knee extensor strength), where we succeeded to test both legs in most participants. Our careful assessment of bone status resulted in the successful inclusion of patients with bone disease in the lower extremities, as long as there was no restriction in one of the legs. Thus, we allow inclusion of patients with bone disease and even patients with assessed increased risk of fracture. Instead of excluding these patients, we differentiate testing and exercising according to bone disease and pain, and therefore, we were able to carry out tests and exercise in a safe manner. In general, other studies excluded patients with risk of fracture [22–24], and only one study specifically defined this risk [25]. Our bone assessment might explain the higher rate of test completion than seen in an earlier study, where 76% completed the isometric strength measurement at the initial assessment, 1–2 weeks after diagnosis [46].

All studies, except Groeneveldt et al. [25], were designed by adapting the program individually at baseline [22–25, 43] and with adjustments during the intervention period, based on the patients’ exercise logs [22–25] or by brief, individual counseling to enhance compliance and motivation [43]. Only Groeneveldt et al. [25] had supervised exercise as part of the intervention, which is important in order to make adjustments and to enhance compliance [7, 47]. We consider the combined exercise intervention (supervised and home-based), a strength for our study.

The effects of exercise on physical parameters, QoL, and fatigue have been conflicting across earlier studies in patients with MM. Suboptimal compliance, the timing of the intervention, or non-optimal intensity are reasons discussed by authors to be possible explanations for the non-significant results [22–24, 43]. Thus, so far, the effectiveness of physical exercise in patients with MM is unclear, which highlights the importance of our ongoing randomized trial.

In conclusion, early initiated, individualized physical exercise in patients with multiple myeloma is feasible and safe, even in older patients and in patients with bone involvement. We succeeded in including an age representative cohort of newly diagnosed patients and in including patients with clinical bone disease. Our ongoing randomized study will hopefully contribute importantly to answer the question if early initiated physical exercise in patients with multiple myeloma is effective on physical function, quality of life, pain, and bone disease.

## Data Availability

The datasets used and/or analyzed during the current study are available from the corresponding author on reasonable request.

## Abbreviations

AEs: Adverse events
CG: Control group
HDT-SCT: High-dose therapy with stem cell support
IG: Intervention group
MM: Multiple myeloma
PS: Performance status
QoL: Quality of life
RCT: Randomized controlled trial
